# Nonlinear Pyroelectric and Photoelectric Responses of GaN Nanowires to Ultraviolet Excitation

**DOI:** 10.3390/ma18061276

**Published:** 2025-03-13

**Authors:** Shikuan Chen, Guoshuai Qin, Zhenyu Wang, Mingkai Guo, Cuiying Fan, Minghao Zhao, Chunsheng Lu

**Affiliations:** 1 School of Mechanics and Safety Engineering, Zhengzhou University, Zhengzhou 450001, China; skc0707@163.com (S.C.); memhzhao@zzu.edu.cn (M.Z.); 2 School of Mechanical and Electrical Engineering, Henan University of Technology, Zhengzhou 450001, China; wangzhenyu962022@163.com (Z.W.); guomk@haut.edu.cn (M.G.); 3 School of Mechanical Engineering, Zhengzhou University, Zhengzhou 450001, China; fancy@zzu.edu.cn; 4 Henan Key Engineering Laboratory for Anti-Fatigue Manufacturing Technology, Zhengzhou University, Zhengzhou 450001, China; 5 School of Civil and Mechanical Engineering, Curtin University, Perth, WA 6845, Australia

**Keywords:** GaN nanowire, pyro-phototronic effect, nonlinearity, ultraviolet light excitation

## Abstract

Gallium nitride (GaN), an advanced piezoelectric semiconductor, shows strong potential for ultraviolet (UV) applications due to its prominent thermoelectric, photoelectric, and mechanoelectrical coupling effects, all of which are critical to device performance. This paper focuses on one-dimensional GaN nanowires and introduces a nonlinear theoretical model to describe pyroelectric and photoelectron effects under UV excitation. The model accounts for both photothermal and photoconductive effects. Using the perturbation method, we derive an approximate analytical solution for the internal physical field of the nanowire under UV light irradiation, which aligns well with the results from nonlinear numerical simulations. Compared to a light intensity of 2 W/m^2^, a light intensity of 6 W/m^2^ leads to a 45% increase in electron concentration, a 235% rise in hole concentration, a 146% increase in potential, and a 274% increase in polarization charge concentration. The pyro-phototronic effect enables UV light to modulate the electrical transport characteristics of a Schottky junction. This study addresses the limitations of linearized models for handling large disturbances, providing a comprehensive theoretical and computational framework for advancing GaN micro- and nanoscale devices and enabling effective, non-contact control.

## 1. Introduction

Piezoelectric semiconductors are a class of intelligent materials that combine piezoelectric and semiconductor properties, enabling interaction between mechanical fields and charge carriers. These materials have significant potential for multifunctional device applications [1]. Recent advancements in fabrication technologies have led to the development of various forms of piezoelectric semiconductors, including fibers, tubular structures, and thin films [2,3,4,5]. Commonly designed as single structures [6,7] or arrays [8], they are widely used in nanogenerators [9,10,11], wearable piezoelectric devices [12,13], and energy conversion systems [14,15], driving the growth of research fields like piezoelectric electronics and piezoelectric optoelectronics [16,17].

Nanowires, as fundamental components of smart electronic devices, play a critical role in various applications. The effective anisotropy and interactions within ferromagnetic nanowire arrays [18,19], the angular dependence of coercive force [20], magneto-optical interactions in magnetic structures [21], and spin-wave propagation in microstrips [22] have all been extensively studied. For piezoelectric semiconductor nanowires, the mechanical behavior of nanowires, such as their performance under tensile [23,24,25], bending [26,27], vibrational stresses [28,29], and doping conditions [30,31], has been extensively studied. However, these studies often rely on linearized assumptions for carrier behavior, which oversimplifies the complex, nonlinear interactions between the piezoelectric effect and charge carriers. While linear models work well for small loads, they fail to accurately capture the behavior of piezoelectric semiconductors under more complex or intense conditions. In addition to mechanical stimuli, piezoelectric semiconductors are capable of transducing thermal [32,33,34], magnetic [35,36,37], and optical signals [38,39] into electrical responses. It is worth noting that, however, most research has focused on the strain-induced electromechanical properties, with limited exploration of non-contact regulation mechanisms, such as photothermal effects.

Introducing optical signals, particularly ultraviolet (UV) light, can excite a substantial number of non-equilibrium carriers in piezoelectric semiconductors. Photon energy not only generates carriers but also raises the material’s temperature, affecting carrier distribution and motion through pyroelectric and thermoelastic piezoelectric coupling mechanisms. Compared to mechanical regulation, this non-contact method of performance control is more direct and efficient. Optically controlled piezoelectric semiconductors are characterized by complex multi-physical field coupling phenomena. While current research primarily focuses on experimental analyses of the external responses of various materials, theoretical studies on the internal field distribution remain limited. A thorough understanding of the internal field distribution is essential for advancing the development and design of piezoelectric semiconductor devices.

In this paper, we focus on the multiphysics coupling model and its nonlinear analytical approach for gallium nitride (GaN) nanowires under UV irradiation. Using a one-dimensional nonlinear theoretical framework for piezoelectric semiconductors, we systematically investigate the behavior of GaN nanowires under UV light excitation. The paper is organized as follows. First, we develop a one-dimensional model that integrates pyroelectric and photoconductive effects under UV excitation. Next, we apply the perturbation method to solve for the distribution of physical fields in GaN nanowires exposed to UV light. We then compare the perturbation-based method with numerical simulations. An analysis is presented on the effects of UV light on the electrical transport properties and control capabilities of Schottky structures. Finally, the key findings are summarized.

## 2. The Coupling Model

In semiconductors, when the energy of an incident photon exceeds the band gap energy, an electron is excited, causing it to jump from the valence band to the conduction band, leaving a hole in the valence band. This process forms an electron–hole pair that contributes to conduction. Furthermore, since the size of the nanowires is smaller than the wavelength of UV light, the effects of reflection and refraction are minimal when the light is uniformly incident on the nanowires. Therefore, in this study, only light absorption is considered. The steady-state concentrations of non-equilibrium carriers under uniform illumination [40] can be expressed as

(1) $$\begin{array}{c} \Delta n_{\mathrm{opt}}=\beta \alpha P_{\mathrm{opt}}\lambda h^{-1}c^{-1}e^{-\alpha d}\tau_{n},\\ \Delta p_{\mathrm{opt}}=\beta \alpha P_{\mathrm{opt}}\lambda h^{-1}c^{-1}e^{-\alpha d}\tau_{p}, \end{array}$$

where *β* is the quantum efficiency, representing the number of photocarrier pairs generated per photon, *α* is the absorption coefficient, and *P*_opt_ and *l* are the illumination intensity and wavelength, respectively. Additionally, *h*, *c*, and *d* denote Planck’s constant, the speed of light, and the incident depth, respectively. The terms *τ_n_* and *τ_p_* refer to the lifetimes of the excited electrons and holes, respectively. The carrier lifetime is influenced by various factors, including recombination mechanisms, material quality, and external electric fields. In this study, we consider an ideal low-defect material, where radiative recombination is the dominant process. Under these conditions, the lifetimes of electrons and holes can be reasonably assumed to be equal.

**Figure 1:**
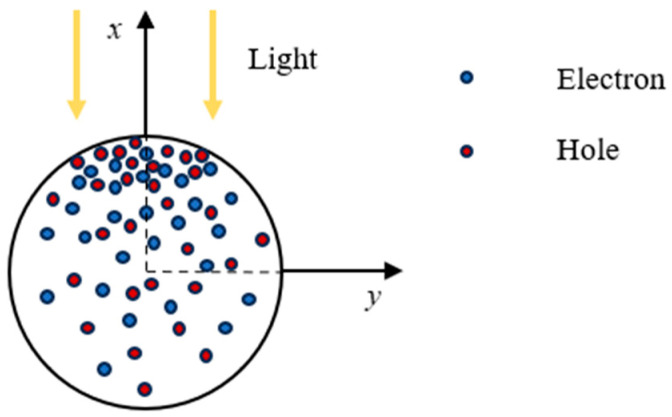
Schematic illustration of photoexcitation carrier attenuation.

As illustrated in Figure 1, photoexcited carriers decay gradually as the incident light penetrates deeper. For a one-dimensional piezoelectric semiconductor with a radius *r*, the average concentration of photogenerated carriers across the cross-section is given by

(2) $$\begin{array}{c} \Delta {\bar{n}}_{\mathrm{opt}}=(\iint_{x^{2}+y^{2}\leq r^{2}}\Delta n_{\mathrm{opt}}dxdy)/\pi r^{2},\\ \Delta {\bar{p}}_{\mathrm{opt}}=(\iint_{x^{2}+y^{2}\leq r^{2}}\Delta p_{\mathrm{opt}}dxdy)/\pi r^{2}. \end{array}$$

In addition to exciting electron–hole pairs, the remaining energy from photon absorption is transferred to the lattice, raising the semiconductor’s temperature. According to the principle of heat balance [41], the rate of temperature change over time is described by

(3) $$C_{s}\frac{d(\theta -\theta_{s})}{dt}+G_{s}(\theta -\theta_{0})=SI,$$

where *C_s_* is the semiconductor’s heat capacity, *G_s_* is the heat exchange coefficient with air, and *θ_s_* and *θ*_0_ are the initial and room temperatures, respectively. *S* is the illuminated area and the excess light intensity is denoted by *I* = (*hv* − *qEg*)/(*hv*)*P*_opt_, with *hv* being the photon energy, *q* the elementary charge, and *Eg* the band gap energy.

At the initial moment (*t* = 0), the temperature change Δ*θ* = 0. Solving the equation yields the expression for temperature change over time, that is

(4) $$\Delta \theta (t)=\theta -\theta_{s}=\Delta \theta_{\mathrm{opt}}(1-e^{-t/t_{\theta }}),$$

where Δ*θ*_opt_ = *SI/G_s_* is the maximum temperature change, and *t_q_* is the thermal time constant. In a steady state, the light time $t\gg \tau_{\theta }$, we have Δ*θ* (*t*) = Δ*θ*_opt_. Temperature variations typically lead to changes in the bandgap, which in turn affect photoexcited carriers. However, as a wide-bandgap material, GaN exhibits minimal reduction in the bandgap with increasing temperature. Since the energy of UV light already exceeds the bandgap of GaN, the influence of temperature on the excitation process is negligible.

**Figure 2:**
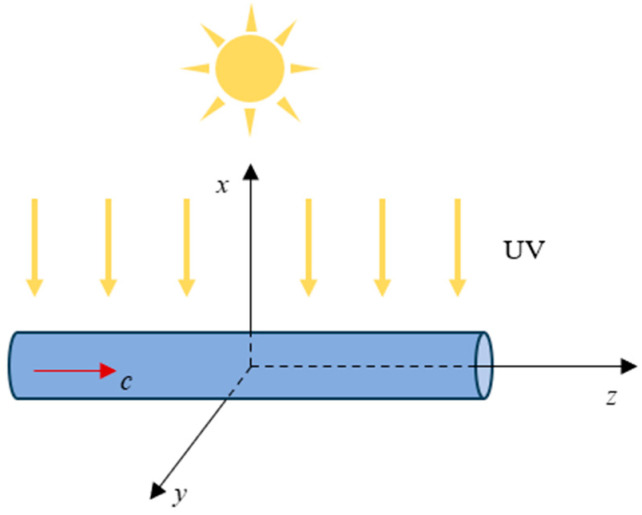
Illustration of the physical model for a GaN nanowire under UV light.

Taking a one-dimensional GaN nanorod with a length of 2*L* as an example (see Figure 2), the physical and mechanical behaviors in a steady state are governed by the motion equation, electrostatics Gauss’s law, and the current continuity equation, that is

(5) $$\begin{array}{l} \frac{\partial \sigma_{zz}}{\partial z}=0,\\ \frac{\partial D_{z}}{\partial z}=q(p-n+N_{D}-N_{A}),\\ \frac{\partial J_{z}^{n}}{\partial z}=0,\\ \frac{\partial J_{z}^{p}}{\partial z}=0, \end{array}$$

where *σ_zz_*, *D_z_*, $J_{z}^{n}$ and $J_{z}^{p}$ are the stress tensor, electric displacement, electron concentration density, and hole current density, respectively. *p*, *n*, *N_D_*, and *N_A_* are the hole concentrations, electron concentrations, ionization degrees of donor, and acceptor impurities under light, respectively.

For a one-dimensional piezoelectric semiconductor polarized along the *z* axis, thermoelastic and pyroelectric effects are considered through the constitutive relationships

(6) $$\begin{array}{l} \sigma_{zz}=c_{33}\varepsilon_{zz}-e_{33}E_{z}-\lambda_{33}\Delta \theta_{\mathrm{opt}},\\ D_{i}=e_{33}\varepsilon_{zz}+\kappa_{33}E_{z}+p_{33}\Delta \theta_{\mathrm{opt}},\\ J_{z}^{n}=qn\mu_{33}^{n}E_{z}+qd_{33}^{n}n_{,z},\\ J_{z}^{p}=qp\mu_{33}^{p}E_{z}+qd_{33}^{p}p_{,z}, \end{array}$$

where *c*_33_, *e*_33_, and *κ*_33_ are the elastic coefficients, piezoelectric coefficients, and dielectric constant, *λ*_3_ and *p*_33_ are the thermal expansion coefficient and pyroelectric coefficient. *e_zz_* is the strain tensor, *E_z_* is the electric field strength. $\mu_{n}^{33}$ and $\mu_{p}^{33}$ denote the electron and hole mobilities, respectively; $d_{n}^{33}$ and $d_{p}^{33}$ are electron and hole diffusion constants, respectively. It can be observed that in the constitutive equation for the current, terms such as $qn\mu_{n}^{33}E_{z}$ and $qp\mu_{p}^{33}E_{z}$, where the product of the unknown carriers and the unknown electric field exhibits strong nonlinearity, contribute to the nonlinear behavior.

The diffusion and mobility of carriers satisfy the Einstein relation, namely,

(7) $$\frac{\mu_{33}^{n}}{d_{33}^{n}}=\frac{\mu_{33}^{p}}{d_{33}^{p}}=\frac{q}{k_{B}T_{0}},$$

where *k_B_* and *T*_0_ are Boltzmann’s constant and reference temperature. The strain *e_zz_* and the electric field *E_z_* are related to the mechanical displacement *u* and the electric potential *φ*, respectively, that is,

(8) $$\begin{array}{l} \varepsilon_{zz}=\frac{\partial u}{\partial z},\\ E_{z}=-\frac{\partial \varphi }{\partial z}, \end{array}$$

where *u* and *φ* are the mechanical displacement and electric potential, respectively.

Substituting Equation (6) into Equation (5), the governing equations are obtained by

(9) $$\begin{array}{l} c_{33}\frac{\partial^{2}u}{\partial z^{2}}+e_{33}\frac{\partial^{2}\varphi }{\partial z^{2}}-\lambda_{33}\frac{\partial \Delta \theta_{opt}}{\partial z}=0,\\ e_{33}\frac{\partial^{2}u}{\partial z^{2}}-\kappa_{33}\frac{\partial^{2}\varphi }{\partial z^{2}}+p_{33}\frac{\partial \Delta \theta_{opt}}{\partial z}=q(p-n+N_{D}-N_{A}),\\ -qn\mu_{33}^{n}\frac{\partial^{2}\varphi }{\partial z^{2}}+qd_{33}^{n}\frac{\partial^{2}n}{\partial z^{2}}=0,\\ -qp\mu_{33}^{p}\frac{\partial^{2}\varphi }{\partial z^{2}}-qd_{33}^{p}\frac{\partial^{2}p}{\partial z^{2}}=0. \end{array}$$

When a piezoelectric semiconductor is illuminated, its internal electromechanical field is governed by Equation (9). By applying the boundary conditions and interface continuity conditions of the actual piezoelectric semiconductor device, the distribution of the internal electromechanical field can be determined.

## 3. Nonlinear Electromechanical Fields

To simplify the solution of the electromechanical field in piezoelectric semiconductors, most studies linearize the carrier concentrations, expressed as *n* = *n*_0_ *+*Δ*n*, *p* = *p*_0_ *+*Δ*p*. However, under large loads, the results from linearized approximations deviate significantly from the true values [42,43,44], necessitating an analysis based on the actual nonlinear constitutive relationships.

The perturbation method is particularly well-suited for addressing complex nonlinear and multiphysics coupling problems. By employing series expansion, this approach allows for the iterative approximation of exact solutions while significantly reducing computational complexity, making it highly efficient for large-scale numerical calculations. A nonlinear perturbation solution to Equation (9) is given by

(10) $$u=\sum_{m=0}^{\infty }u^{\left(m\right)},\varphi =\sum_{m=0}^{\infty }\varphi^{\left(m\right)},n=\sum_{m=0}^{\infty }n^{\left(m\right)},p=\sum_{m=0}^{\infty }p^{\left(m\right)}.$$

Under UV irradiation, and there is no current flow in and out, the boundary conditions at both ends of the nanowires can be written as

(11) $$\begin{array}{l} \sigma_{z}(\pm L)=0,\\ D_{z}(\pm L)=0,\\ J_{z}^{n}(\pm L)=0,\\ J_{z}^{p}(\pm L)=0. \end{array}$$

From Equations (5) and (11), we can get $J_{z}^{n}=0,J_{z}^{p}=0$.

Substituting Equation (10) into Equation (9) under the conditions of $J_{z}^{n}=0,J_{z}^{p}=0$, the first-order perturbed governing equations are obtained as

(12) $$\begin{array}{l} c_{33}\frac{\partial^{2}u^{\left(1\right)}}{\partial z^{2}}+e_{33}\frac{\partial^{2}\varphi^{\left(1\right)}}{\partial z^{2}}=0,\\ e_{33}\frac{\partial^{2}u^{\left(1\right)}}{\partial z^{2}}-\kappa_{33}\frac{\partial^{2}\varphi^{\left(1\right)}}{\partial z^{2}}=q(p^{\left(1\right)}-n^{\left(1\right)}),\\ -qn^{\left(0\right)}\mu_{33}^{n}\frac{\partial \varphi^{\left(1\right)}}{\partial z}+qd_{33}^{n}\frac{\partial n^{\left(1\right)}}{\partial z}=0,\\ -qP^{\left(0\right)}\mu_{33}^{n}\frac{\partial \varphi^{\left(1\right)}}{\partial z}+qd_{33}^{n}\frac{\partial P^{\left(1\right)}}{\partial z}=0. \end{array}$$

Then, a differential equation about the potential *j* can be written as

(13) $$\frac{\partial^{3}\varphi^{\left(1\right)}}{\partial z^{3}}-k^{2}\frac{\partial \varphi^{\left(1\right)}}{\partial z}=0,$$

where *k*^2^ = *q*(*n*^(0)^ *+ p*^(0)^)*V_th_/ε_33_*, *V_th_* = *q/k_B_T*_0_, and *ε*_33_ = *κ*_33_ + *e*_33_^2^*/c*_33_. Solving Equation (13) yields a general solution to the potential, that is,

(14) $$\varphi^{\left(1\right)}=C_{1}\cosh kz+C_{2}\sinh kz+C_{3}.$$

Further, based on Equation (12), the general solutions of *u*^(1)^ and *n*^(1)^ are

(15) $$u^{\left(1\right)}=-\frac{e_{33}}{c_{33}}\varphi^{\left(1\right)}+C_{4}z+C_{5},$$

(16) $$n^{\left(1\right)}=n^{\left(0\right)}V_{th}\varphi^{\left(1\right)}+C_{6},$$

where *C_x_* (*x* = 1, 2, …, 6) are the integral constants.

From Equations (12) and (16), the general solution of *p*^(1)^ is

(17) $$p^{\left(1\right)}=n^{\left(1\right)}-\frac{\varepsilon_{33}}{q}\frac{\partial^{2}\varphi^{\left(1\right)}}{\partial z^{2}}.$$

Here, *n*^(1)^ and *p*^(1)^ satisfy the electrical neutral conditions, that is,

(18) $$\int_{-L}^{L}p^{\left(1\right)}dz=0,\int_{-L}^{L}n^{\left(1\right)}dz=0.$$

At the position of *z* = 0, the potential and displacement are

(19) $$u^{\left(1\right)}=0,\varphi^{\left(1\right)}=0.$$

Substituting Equations (14)–(17) into Equations (10), (18), and (19), *C_x_* (*x* = 1, 2, …, 6) can be solved and we have

(20) $$\begin{array}{lll} C_{1}=0, & C_{2}=0, & C_{3}=\frac{e_{33}\lambda_{33}\theta +c_{33}p_{33}\theta }{\kappa \varepsilon_{33}c_{33}\cosh\kappa L},\\ C_{4}=\frac{\lambda_{33}\theta }{c_{33}}, & C_{5}=0, & C_{6}=0. \end{array}$$

Thus, the analytical expressions for each internal electromechanical field based on the first-order perturbation solution are

(21) $$\begin{array}{l} \varphi^{\left(1\right)}=\frac{e_{33}\lambda_{33}\theta +c_{33}p_{33}\theta }{\kappa \varepsilon_{33}c_{33}\cosh\kappa L}\sinh\kappa z,\\ u^{\left(1\right)}=\frac{e_{33}^{2}\lambda_{33}\theta +e_{33}c_{33}p_{33}\theta }{\kappa \varepsilon_{33}c_{33}^{2}\cosh\kappa L}n^{\left(0\right)}V_{th}\sinh\kappa z+\frac{\lambda_{33}\theta }{c_{33}}z,\\ n^{\left(1\right)}=\frac{e_{33}\lambda_{33}\theta +c_{33}p_{33}\theta }{\kappa \varepsilon_{33}c_{33}\cosh\kappa L}n^{\left(0\right)}V_{th}\sinh\kappa z,\\ p^{\left(1\right)}=\frac{e_{33}\lambda_{33}\theta +c_{33}p_{33}\theta }{\kappa \varepsilon_{33}c_{33}\cosh\kappa L}p^{\left(0\right)}V_{th}\sinh\kappa z. \end{array}$$

The polarization charges generated inside the GaN nanowire can be expressed as

(22) $$\rho^{\left(1\right)}=-e_{33}\frac{\partial^{2}u^{\left(1\right)}}{\partial z^{2}}+(\kappa_{33}-\varepsilon_{0})\frac{\partial^{2}\varphi^{\left(1\right)}}{\partial z^{2}}.$$

The first-order perturbation solution may lack sufficient accuracy under large loads. Therefore, we proceed to derive the second-order perturbation solution. The second-order perturbed governing equation can be obtained as

(23) $$\begin{array}{l} c_{33}\frac{\partial^{2}u^{\left(2\right)}}{\partial z^{2}}+e_{33}\frac{\partial^{2}\varphi^{\left(2\right)}}{\partial z^{2}}=0,\\ e_{33}\frac{\partial^{2}u^{\left(2\right)}}{\partial z^{2}}-\kappa_{33}\frac{\partial^{2}\varphi^{\left(2\right)}}{\partial z^{2}}=q(p^{\left(2\right)}-n^{\left(2\right)}),\\ -qn^{\left(0\right)}\mu_{33}^{n}\frac{\partial \varphi^{\left(2\right)}}{\partial z}-qn^{\left(1\right)}\mu_{33}^{n}\frac{\partial \varphi^{\left(1\right)}}{\partial z}+qd_{33}^{n}\frac{\partial n^{\left(2\right)}}{\partial z}=0,\\ -qp^{\left(0\right)}\mu_{33}^{n}\frac{\partial \varphi^{\left(2\right)}}{\partial z}-qp^{\left(1\right)}\mu_{33}^{n}\frac{\partial \varphi^{\left(1\right)}}{\partial z}+qd_{33}^{n}\frac{\partial p^{\left(2\right)}}{\partial z}=0. \end{array}$$

The boundary condition of the second-order perturbed governing equations are

(24) $$\begin{array}{ll} T^{\left(2\right)}(\pm L)=0, & D^{\left(2\right)}(\pm L)=0,\\ \int_{-L}^{L}n^{\left(2\right)}dz=0, & \int_{-L}^{L}p^{\left(2\right)}=0,\\ u^{\left(2\right)}(0)=0, & \varphi^{\left(2\right)}(0)=0. \end{array}$$

The solution process for the second-order perturbation equation is similar to that for the first-order equation. Its solution is given by

(25) $$\begin{array}{ll} \varphi^{\left(2\right)}= & \frac{qm^{2}(n^{\left(0\right)}-p^{\left(0\right)})(1-2\cosh\kappa L+\cosh\kappa z)\sinh^{2}\frac{\kappa z}{2}}{3\varepsilon_{33}\kappa^{2}},\\ u^{\left(2\right)}= & \frac{qm^{2}e_{33}(n^{\left(0\right)}-p^{\left(0\right)})(1-2\cosh\kappa L+\cosh\kappa z)\sinh^{2}\frac{\kappa z}{2}}{3c_{33}\varepsilon_{33}\kappa^{2}},\\ n^{\left(2\right)}= & \frac{-8qm^{2}n^{\left(0\right)}\kappa L(n^{\left(0\right)}-p^{\left(0\right)})V_{th}\cosh\kappa L\cosh\kappa z}{24\varepsilon_{33}\kappa^{3}L}+\\ & \frac{2qm^{2}n^{\left(0\right)}\kappa L(3\varepsilon_{33}\kappa^{2}+q(n^{\left(0\right)}-p^{\left(0\right)})V_{th})\cosh2\kappa z}{24\varepsilon_{33}\kappa^{3}L}-\\ & \frac{3qm^{2}n^{\left(0\right)}(\varepsilon_{33}\kappa^{2}+q(n^{\left(0\right)}-p^{\left(0\right)})V_{th})\sinh2\kappa z}{24\varepsilon_{33}\kappa^{3}L},\\ p^{\left(2\right)}= & \frac{-8qm^{2}p^{\left(0\right)}(8\kappa L(n^{\left(0\right)}-p^{\left(0\right)}))V_{th}\cosh\kappa L\cosh\kappa z}{24\varepsilon_{33}\kappa^{3}L}+\\ & \frac{2qm^{2}p^{\left(0\right)}\kappa L(3\varepsilon_{33}\kappa^{2}+q(n^{\left(0\right)}-p^{\left(0\right)}))V_{th}\cosh\kappa L\cosh\kappa z}{24\varepsilon_{33}\kappa^{3}L}-\\ & \frac{3qm^{2}p^{\left(0\right)}(\varepsilon_{33}\kappa^{2}+q(n^{\left(0\right)}-p^{\left(0\right)})V_{th})\sinh2\kappa z}{24\varepsilon_{33}\kappa^{3}L},\\ \rho^{\left(2\right)}= & -e_{33}\frac{\partial^{2}u^{\left(2\right)}}{\partial z^{2}}+(\kappa_{33}-\varepsilon_{0})\frac{\partial^{2}\varphi^{\left(2\right)}}{\partial z^{2}}, \end{array}$$

where

(26) $$\begin{array}{l} m=\frac{e_{33}\lambda_{33}\theta +c_{33}p_{33}\theta }{\kappa \varepsilon_{33}c_{33}\cosh\kappa L}V_{th},\\ \theta =SG_{s}^{-1}(p_{opt}-qE_{g}h^{-1}c^{-1}\lambda p_{opt}),\\ n^{\left(0\right)}=N_{D}^{+}+\eta_{n}\lambda p_{opt},\\ p^{\left(0\right)}=N_{A}^{-}+\eta_{P}\lambda p_{opt}. \end{array}$$

## 4. Electromechanical Field Under UV Irradiation

**Figure 3:**
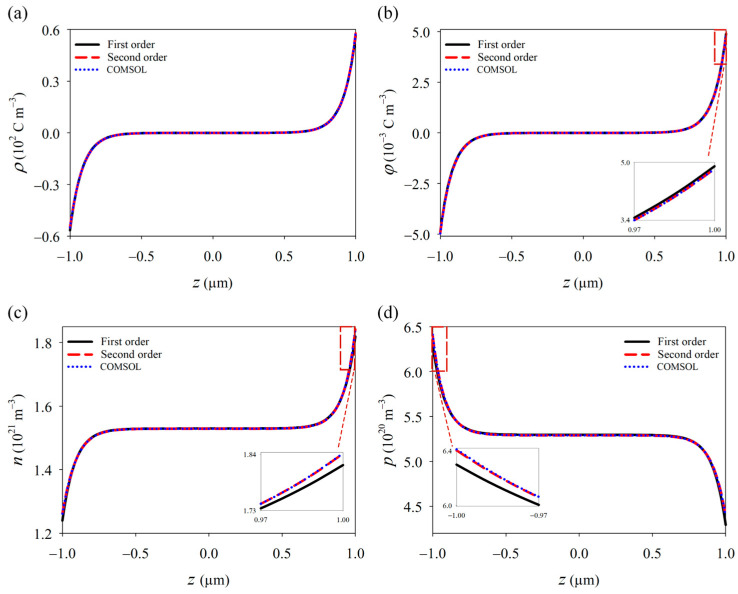
Perturbation solutions of (**a**) polarization charge, (**b**) potential, (**c**) electron, and (**d**) cavity with COMSOL numerical solutions.

Using the perturbation solution, we analyze a GaN nanowire with a length of 2 μm and a diameter of 20 nm under UV light excitation at a wavelength of 210 nm. The initial carrier concentration is *N_D_* = 10^21^ m^−3^. The relevant material constants were taken from previous studies [24,45,46]. We selected a UV light intensity of 6 Wm^−2^ and utilized the PDE module of COMSOL Multiphysics software (version 6.2) to perform nonlinear numerical iterations. The model was divided into 2500 elements, with a calculation time of one minute. The results obtained are compared with the first-order and second-order solutions to verify the accuracy of the perturbation method (see Figure 3).

The results show discrepancies between the first-order perturbation solution (linear solution) and the nonlinear numerical simulations, especially in terms of electron and hole concentrations. In contrast, the second-order perturbation solution closely matches the nonlinear numerical results. Therefore, for device design, the perturbation solution can be used for preliminary evaluation, while numerical simulations should be employed for verification, ensuring both efficiency and accuracy in practical applications.

**Figure 4:**
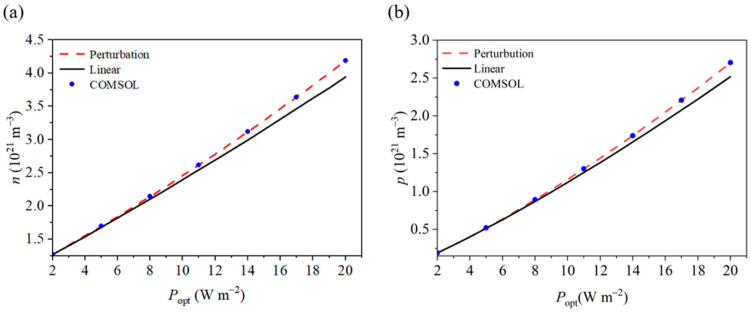
Perturbation solutions, linear solutions, and COMSOL numerical solutions for (**a**) electrons and (**b**) holes at the ends of nanowires.

We further analyzed the difference between the linear and nonlinear solutions by examining the variations in carrier concentrations at the end of the nanowire under different light intensities (see Figure 4). As the light intensity increases, the discrepancy between the linear and nonlinear solutions also grows. At a light intensity of 2 Wm^−2^, the difference in electron concentration is 0.2%, and the difference in hole concentration is 0.3%. When the light intensity increases to 20 Wm^−2^, the difference in electron concentration rises to 5.8% (see Figure 4a), and the difference in hole concentration increases to 6.9% (see Figure 4b). Therefore, under higher light intensities, the accuracy of the linear solution diminishes, while the perturbed solution maintains high accuracy. When designing piezoelectric semiconductor devices, nonlinear analysis can effectively meet the requirements under small loads and provide enhanced accuracy under large loads, reflecting the true operational status of the device. This method aids in the design of more efficient and widely applicable devices, enables performance prediction at high carrier concentrations, and improves device reliability.

**Figure 5:**
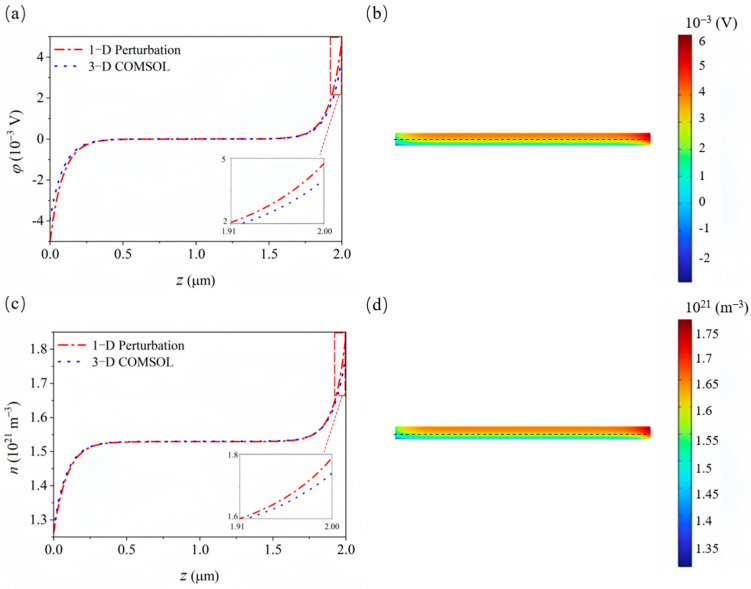
The 1-D perturbation solutions of (**a**) potential and (**c**) electron, with 3-D COMSOL numerical solutions; (**b**,**d**) are their corresponding cloud pictures.

In most device designs, piezoelectric semiconductor nanowires are typically treated as one-dimensional structures; however, this approach overlooks the effects in the other directions. Therefore, we further analyzed the electromechanical electric field distribution of three-dimensional GaN along the axis at a light intensity of 6 Wm^−2^ to validate the accuracy of the one-dimensional analysis (see Figure 5). Both ends are simply supported, with no current entering or exiting at the boundaries, and the initial carrier concentration is *N_D_* = 10^21^ m^−3^. The cloud map indicates that when three-dimensional GaN is exposed to light, the changes in potential and carrier distribution are not limited to the ends but exhibit an overall antisymmetric pattern (see Figure 5b,d). This occurs because the uniform temperature increase in GaN upon light exposure induces pyroelectric effects in the radial direction, leading to the distribution of polarized charges along the radial axis. Consequently, the potential and carriers within the material are redistributed. A significant difference in carrier concentration is observed only at one end, as the overall change in carrier density is similar. However, due to the redistribution induced by polarized charges, more carriers accumulate at the surface, resulting in a lower concentration along the axis. Although there are discrepancies between the one-dimensional perturbation solution and the three-dimensional calculation results in terms of potential and carrier distribution at both ends of the axis, the one-dimensional perturbation solution can still provide a relatively fast prediction of the properties of piezoelectric semiconductors.

**Figure 6:**
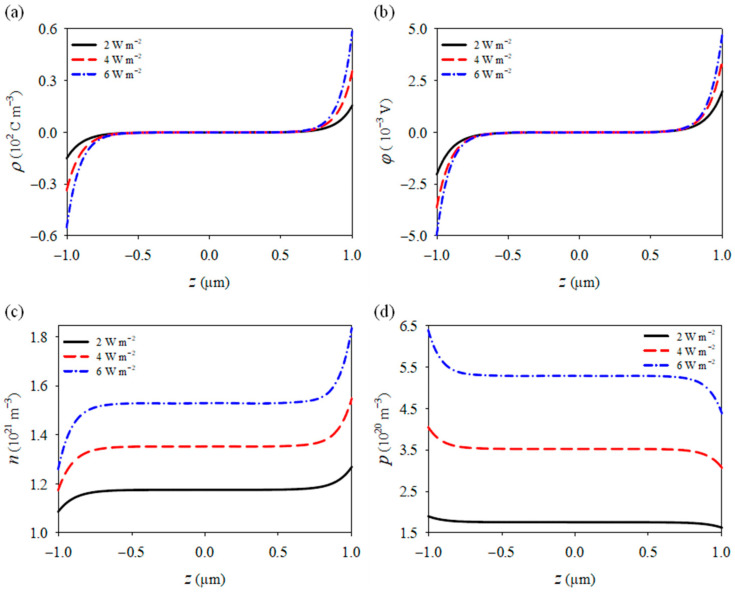
Distribution of (**a**) polarized charges, (**b**) potentials, (**c**) electrons, and (**d**) holes under light loads.

Further investigation revealed the effects of varying light intensities on the physical fields of GaN nanowires (see Figure 6). Under high-energy UV irradiation, GaN absorbs photon energy, resulting in a substantial increase in photogenerated carriers within the material. Beyond optical excitation, photons also impart excess energy that induces lattice vibrations, raising the temperature of the material. This thermal effect causes the relative displacement of positive and negative charges within GaN, generating pyroelectric charges through the photoinduced pyroelectric effect. As a result, polarized charges of opposite polarity accumulate at both ends of the nanowire (see Figure 6a), generating a positive potential at the right end and a negative potential at the left end (see Figure 6b). Under the influence of these potentials, electrons are redistributed: the positive potential at the right end attracts electrons, increasing their concentration on that side, while the negative potential at the left end repels electrons, decreasing their concentration on the left side. This causes the electron distribution to align with the electric potential trend, while the hole distribution exhibits the opposite behavior (see Figure 6c,d). In piezoelectric semiconductor devices, the charges accumulated on the surface are collected by external circuits and converted into electrical signals, which are then processed and amplified by the system to extract information related to external physical quantities.

As the light intensity increases, the changes in polarized charges, potential, and carrier concentrations at both ends also become more pronounced. Compared to a light intensity of 2 Wm^−2^, the electron concentration increases by 45%, the hole concentration rises by 235%, the potential grows by 146%, and the polarization charge concentration increases by 274%. This demonstrates that light intensity can significantly affect the internal field distribution in piezoelectric semiconductor devices. The increase in carrier concentration and the enhancement of the internal electric field leads to a stronger electric field gradient, which accelerates carrier drift, improves energy conversion efficiency, and enhances signal output.

**Figure 7:**
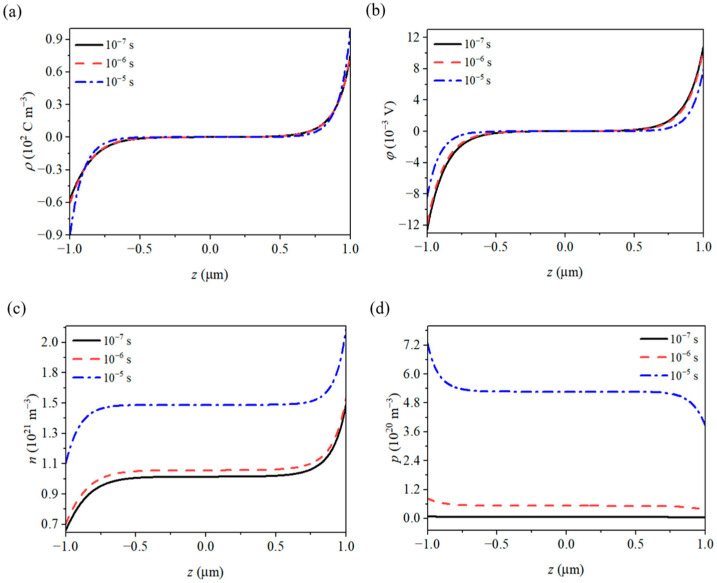
Distributions of (**a**) polarized charges, (**b**) potentials, (**c**) electrons, and (**d**) holes under different photoexcited carrier lifetimes.

The lifetime of photogenerated carriers is influenced by various factors, including material properties, morphology, surface effects, and size effects. To further investigate this, we examined the changes in the electromechanical field within GaN nanowires for different carrier lifetimes (see Figure 7). As the carrier lifetime increases, the number of internal carriers also rises. Under the influence of the potentials at both ends, more carriers migrate toward the ends, which increases the electric field gradient. This, in turn, enhances the internal electric field response and leads to an increase in the polarization charge density at both ends. Additionally, the carrier current will neutralize the polarized charges at both ends, leading to a decrease in the potential at those locations.

When the carrier lifetime is 10^−6^ s or 10^−7^ s, the internal physical field of the nanowire changes minimally. However, when the carrier lifetime reaches 10^−5^ s, the physical field experiences significant changes. The polarized charge increases by 30.4%, the potential decreases by 26.7%, the electron concentration rises by 35.8%, and the hole concentration surges by 11,193%. This is because, as the carrier lifetime increases, the number of photogenerated carriers gradually increases, eventually accounting for a larger proportion of the total carrier concentration, which, in turn, significantly affects the internal physical field of the nanowire.

## 5. I-V Characteristics Under UV Irradiation

Schottky-type devices, due to the presence of their built-in electric field, facilitate the separation and transport of photogenerated carriers, which endows them with advantages such as fast response speed and high sensitivity. Taking the Schottky (Ag-GaN) Ohmic contact as an example, the boundary conditions are given by

(27) $$\begin{array}{ll} \sigma_{z}(\pm L)=0, & u(0)=0,\\ V(-L)=V_{a}+V_{b}, & V(L)=0,\\ J_{z}^{n}(-L)=-q\nu_{\mathrm{rec}}^{n}(n-n_{m}), & J_{z}^{p}(-L)=-q\nu_{\mathrm{rec}}^{p}(p-p_{m}),\\ n(L)=N_{D}, & P(L)=N_{A}, \end{array}$$

where *V*_a_ is bias voltage and the effective built-in electric field *V*_b_ = *V*_bi_ *+ V*_ph_ *+ V*_pyro_. *V*_bi_ is the built-in voltage without load. *V*_ph_ and *V*_pyro_ are the potential differences between the two ends caused by photogenerated carriers and pyro-charges, respectively. $v_{\mathrm{rec}}^{n}$ and $v_{\mathrm{rec}}^{p}$ denote the thermal recombination velocities of electrons and holes at the Schottky interface, respectively. *n*_m_ and *p*_m_ are the critical electron and hole concentrations, calculated by

(28) $$\begin{array}{l} n_{m}=N_{c}e^{-\phi_{B}/k_{B}T},\\ p_{m}=n_{i}^{2}/n_{m}, \end{array}$$

where *ϕ_B_* is the GaN surface barrier when the electron energy is equal to the Fermi level, *T* is the absolute temperature, and *N_c_* is the effective density of states of conduction bands.

The Schottky contacts barrier is

(29) $$q\phi_{B}=q\phi_{M}-q\chi ,$$

where *qϕ_M_* = 4.26 eV is work function of Ag, and *qχ* = 4.1 eV is the electron affinity of GaN.

The built-in voltage *V*_bi_ is defined by

(30) $$V_{bi}=\phi_{B}-k_{B}T/q\ln(N_{c}/n_{0}),$$

where *N_C_* = 2.23 × 10^24^ denotes the effective density of states of conduction bands.

**Figure 8:**
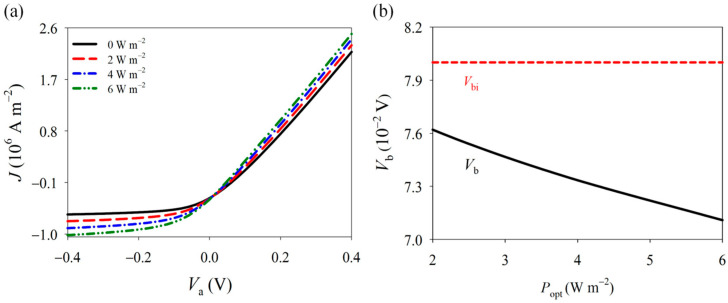
(**a**) I-V characteristics and (**b**) Schottky built-in voltage under different light loads.

Given the typical nonlinear behavior of Schottky junctions, we utilized the PDE module in COMSOL to carry out nonlinear numerical iterations. The model was divided into 2500 elements, with a calculation time of two minutes. Under the excitation of UV light, the current density of the nanowire increases significantly, and the IV characteristics of its Schottky junction are also considerably altered (see Figure 8a). Under bias voltage, GaN nanowires exhibit distinct rectifying characteristics due to the presence of Schottky barriers. However, as the light intensity increases, the turn-on voltage of the Schottky junction decreases, and the current density increases, resulting in a weakened rectifying effect. This phenomenon is caused by the combined action of the photoinduced pyroelectric effect, photoconductive effect, and carrier shielding effect. The large number of photogenerated carriers produced by optical excitation significantly increases the carrier concentration within the nanowire, thereby enhancing its conductivity. Furthermore, although the polarization charges generated by the photoinduced pyroelectric effect can increase the Schottky barrier, the abundance of photogenerated carriers neutralizes the space charge within the Schottky junction, ultimately reducing the Schottky barrier (see Figure 8b). Reducing the height of the Schottky barrier lowers the energy barrier for carriers to cross from the metal into the nanowire, resulting in a larger conduction current. This, in turn, reduces the conduction resistance and power consumption of the piezoelectric semiconductor device. Additionally, a lower barrier height decreases the electron transit time, thereby enhancing the device’s switching speed. As a result, optical control, being an efficient non-contact control method, holds great potential for widespread use in various types of piezoelectric semiconductor devices.

## 6. Conclusions

In this paper, we have analyzed the distribution of electromechanical fields in GaN nanowires under UV irradiation using a one-dimensional nonlinear theoretical framework for piezoelectric semiconductors. The main findings are summarized as follows:

(1) A thermal pyroelectric-photovoltaic model was developed for GaN nanowires under UV excitation, incorporating both photoconductive and pyroelectric effects. Using the perturbation method, an approximate analytical solution for the internal physical field of the nanowire is derived. This solution maintains high accuracy even under large loads, providing a robust theoretical framework for designing piezoelectric semiconductor devices with broader applicability and higher precision.
(2) Due to the pyroelectric-photoelectron effect, UV light induces the redistribution of physical fields within the nanowire. As the light intensity increases, the variations in polarized charges, potential, and carrier concentrations become more pronounced, highlighting the regulatory effect of light on piezoelectric semiconductors. Additionally, the lifetime of photoexcited carriers significantly influences the concentration of photogenerated carriers. When the concentration of photogenerated carriers is low, its impact on the physical fields is minimal. However, as the concentration approaches the initial carrier concentration, it causes substantial changes in the physical fields.
(3) The UV light reduces the Schottky barrier, facilitating the entry of carriers from the metal into the nanowire. This decreases the on-resistance and power consumption of the piezoelectric semiconductor device, accelerates its switching speed, and provides an efficient non-contact method for regulating the device’s electrical transport characteristics.

## Data Availability

The original contributions presented in this study are included in the article. Further inquiries can be directed to the corresponding author.
